# Post-COVID syndrome: pulmonary complications

**DOI:** 10.3906/sag-2106-238

**Published:** 2021-12-17

**Authors:** Dorina ESENDAĞLI, Aydın YILMAZ, Şule AKÇAY, Tevfik ÖZLÜ

**Affiliations:** 1 Department of Chest Diseases, Faculty of Medicine, Başkent University, Ankara Turkey; 2 Department of Chest Diseases, Atatürk Chest Diseases and Thoracic Surgery Centre, Health Sciences University, Ankara Tukey; 3 Department of Chest Diseases, Faculty of Medicine, Karadeniz Technical University, Trabzon Turkey

**Keywords:** Post-COVID, pulmonary complications, multisystem inflammatory syndrome in adults (MIS-A)

## Abstract

Severe acute respiratory syndrome coronavirus 2 (SARS-CoV-2) has infected millions of people worlwide and caused a pandemic that is still ongoing. The virus can cause a disease named as COVID-19, which is composed of multi systemic manifestations with a pulmonary system predominance. As the time passes, we are dealing more and more with a wide variety of effects and complications of the disease in survivors as far as with concerns about the clinical outcome and the timeline of symptoms in different patients. Since the lungs are the most involved organs and the post-COVID prolonged and persistent effects are mainly related to the pulmonary system, it is crucial to define and predict the outcome and to determine the individuals that can progress to fibrosis and loss of function of lungs. This review summarizes the current literature regarding the pulmonary complications in post-COVID syndrome and the management of these conditions.

## 1. Introduction

Our learning process about the COVID-19 disease, first observed in Wuhan city of Hubei province of China since the beginning of December 2019 and announced to the world by the WHO China Office on 31 December 2019 and whose agent was identified on 7 January 2021, still continues. The long-term effects and complications are better understood day by day. After being discharged from the hospital, some symptoms such as cough, exertional dyspnea, muscle aches, and fatigue are observed, sometimes lasting for months, especially in some of the patients who have experienced severe COVID-19. Chronic respiratory failure and permanent lung fibrosis may develop in patients who have been intubated with acute respiratory distress syndrome (ARDS) and received invasive mechanical ventilation (IMV) therapy. Non-pulmonary systemic conditions such as diabetes mellitus, neuromuscular problems, and cardiac pathologies are also experienced. In this article, in the light of current literature, long-term follow-up of COVID-19 patients and the management of the problems observed in this process will be reviewed in terms of post-COVID pulmonary complications. Currently, the best known and most common post-COVID syndrome (PCS) is pulmonary involvement. In this section, this important issue will be reviewed and discussed into six subsections, namely, post-COVID periods and characteristics, post-COVID syndrome definition pathogenesis from postacute COVID to organ dysfunction, postconcussion symptoms (PCS) clinical features, multisystem inflammatory syndrome in adults (MIS-A), and lung complications management in PCS.

## 2. Post-COVID periods and characteristics

### 2.1. COVID-19: clinical course and categorization

Siddiqi HK et al. proposed a 3-stage grading system for the symptomatic disease of COVID-19 including the increasing severity corresponding to different clinical manifestations, response to treatment, and clinical course [1]. Accordingly, the first stage (mild/early infection) is characterized by mild nonspecific symptoms such as fever, malaise and dry cough following infection and incubation. The virus replicates mainly focused on the respiratory system. At this stage, symptomatic treatment is essential. Trial treatments with some antivirals have been applied to prevent progression, reduce transmission, and accelerate recovery. Most patients recover in this stage [1].

The second stage (moderate/pulmonary involvement stage: stage IIa without hypoxia, stage IIb with hypoxia) is characterized by viral pneumonia. The virus continues to replicate and settles in the lungs. Fever, cough, hypoxia, radiological infiltrates, lymphopenia, and acute inflammatory markers are elevated. Most patients at this stage require hospitalization and treatment. Antivirals are used for trial purposes. In the absence of hypoxia (Stage IIa), corticosteroid use should be avoided. When hypoxia develops (Stage IIb), corticosteroid and oxygen therapies are provided [1].

Extrapulmonary systemic hyperinflammation is observed in the third stage (severe/systemic hyperinflammation stage) in a small number of patients. IL-2, IL-6, IL-7, GCSF, macrophage inflammatory protein 1-alpha, TNF-alpha, CRP, ferritin, D-dimer are increased. Troponin and pro-BNP may also be elevated. Shock, vasoplegia, respiratory failure, cardiopulmonary collapse may develop. Systemic involvements such as myocarditis may occur. At this stage, immunomodulatory treatments (corticosteroid, tocilizumab (IL-6 inhibitor) or anakinra (IL-1 receptor antagonist), intravenous immune globulin) are used to suppress systemic inflammation and prevent multiorgan failure that may develop afterwards [1].

### 2.2. COVID-19 timeline

Patients become symptomatic 2–14 days after exposure (mean time: day 5). It usually gets better in 7–10 days or it starts to get worse 7 days after the onset of symptoms, often at day 9–10. Intensive care unit (ICU) support may be required and death usually occurs 14–28 days after the onset of symptoms. In symptomatic, severe cases, symptoms may last up to 6 months after recovery. Permanent sequelae and pulmonary fibrosis may develop in patients who have been treated in the ICU, who have developed acute respiratory distress syndrome (ARDS) and who have undergone IMV. Poor prognostic factors reported in patients are as follows: advanced age, comorbidities, smoking, obesity, genetic predisposition, pregnancy, excessive increase in inflammatory markers, lymphopenia, and treatment-resistant hypoxemia [2–6].

## 3. Post-COVID syndrome (PCS) definition

According to the definitions used in the NICE guideline that includes the acute and chronic stages of the disease, the symptoms are listed below: 

Acute COVID-19: Presence of signs and symptoms of COVID-19 from onset to 4 weeks. Ongoing symptomatic COVID-19: Signs and symptoms of COVID-19 between 4 and 12 weeks.

 Post-COVID-19: Signs and symptoms lasting more than 12 weeks and not explained by an alternate diagnosis.

 Long COVID-19: Refers to the association of both ongoing and post-COVID-19 [7].

When diagnosing post-COVID-19 the following criteria are taken into account: the patient’s symptoms that started in the acute period persist, worsen, recur, or new symptoms develop; worsening, loss of quality of life and functional status happens when compared to pre-COVID-19 and the presence of permanent or progressive radiological pathologies and abnormal laboratory findings in the lungs [8].

However, there are many confounding situations that make the definition of post-COVID-19 difficult. It is not easy to define a timeline for acute and chronic symptoms. It is also difficult to distinguish between post-COVID-19 and the previously known post-intensive care (malnutrition, immobility, anxiety, etc.), post-ARDS, post-mechanical ventilation (barotrauma, fibrosis, pneumothorax, etc.) and post-intubation complications (tracheal trauma, edema, stenosis, etc.). In order to distinguish between previous comorbidities and the effects of COVID-19, it is necessary to know the patient’s pre-COVID-19 baseline status. It is important to distinguish if the clinical outcome is due to post-COVID-19 or post viral infection complications (bronchial hypersensitivity, constitutional problems, etc.), or whether it is a post-secondary infection (resistant bacterial, fungal infections, etc.). Problems after corticosteroid use (muscle weakness, diabetes mellitus (DM), osteoporosis, infections, adrenal suppression, etc.), post immunosuppression effects (anti-IL-6/1), post traumatic syndrome (anxiety, panic, etc.), post thrombotic (myocardial infarction (MI), stroke), pulmonary embolism (PE), etc.), and post-ischemic (infarction, functional limitation, etc.) conditions should also be considered.

### 3.1. Frequency of postconcussion symptoms (PCS)

In a study in the USA, it was shown that only 6.75% of COVID-19 patients returned to their previous health level 14–21 days after a positive test result [9]. Again, of the 233 cases of COVID-19 in the USA, most of which were mild and only 8 patients were hospitalized, 43.4% were shown to have symptoms lasting longer than 30 days, and 24.1% still had at least one symptom after 90 days. When compared to more severe COVID-19 cases, these rates were higher: 59.4% at 30 days and 40.6% at 90 days. In the acute period, complications persisted for 30 days or longer in 14.3% of even mild and asymptomatic cases [10]. It was revealed that 87.4% of COVID-19 patients in Italy still complained of at least one symptom 60 days after discharge [11]. Approximately 10%–20% of patients hospitalized with the diagnosis of COVID-19 required rehospitalization after discharge [12–13]. Of the 47,780 patients discharged from hospital after acute COVID-19 in the UK, who were followed-up at an average of 140 days, around a third of individuals were readmitted (in total 14,060) and more than 1 in 10 (in total 5875) died after discharge [14].

Patients at high risk of long-term complications were treated in ICU or care units, received long-term high-flow oxygen therapy (HFO), non-invasive mechanical ventilation (NIMV, CPAP, BIPAP) and were discharged on oxygen therapy [15].

### 3.2. Importance of PCS

The rationale behind the long-term follow-up of patients treated with a diagnosis of COVID-19 consists of identification and monitorization of the early, medium, and long-term respiratory complications; early detection and treatment of life-limiting complications such as pulmonary fibrosis and pulmonary vascular disease; assessment of patients’ dyspnea, oxygen requirement, provision of rehabilitation, palliative care, symptom management and psychosocial support; ensure radiological resolution of clinically recovered patients and identify and manage previous undiagnosed respiratory disease. This detailed evaluation is recommended to be done after every contact with the patient to detect the complications in the early period and manage permanent symptoms in the future [15].

We still do not have clear information on the optimal timing of the follow-up of these patients. The follow-up period and intervals may vary from patient to patient. Follow-up planning will change according to the severity of the acute disease, the age of the patient, and the accompanying diseases. Young, non-comorbid and outpatient, mild patients who recover without treatment or hospitalization may not require routine follow-up unless they request. In patients who are elderly and have comorbidities and moderate to severe disease but do not require hospitalization should be given an appointment 3 weeks after the onset of the disease. In more severe cases treated at the hospital, the first visit is recommended as soon as within 1 week after discharge or 2–3 weeks at the latest.

In the controls of the cases who have had the COVID-19 disease, first of all, detailed anamnesis is taken and physical examinations are performed in order to determine the current symptoms and signs. Subsequently, their COVID-19 disease histories (time of getting sick, severity of the disease, treatments received, place and duration of treatment, complications, etc.) are questioned. Comorbid diseases, chronic medical conditions and the treatments they are receiving are assessed. The range of laboratory tests and imaging together with functional measurements can be planned according to the clinical findings. Appropriate medical treatments are arranged for the existing symptoms or comorbid chronic conditions and a plan is made for a regular follow-up. If worsening of symptoms occurs, the patients should be recommended to come earlier and not wait for the next visit. Education is provided on possible symptoms after persistent COVID-19 and the need for rehabilitation is evaluated [8].

In the light of the information we have obtained from the community-acquired pneumonia, patients diagnosed with COVID-19 pneumonia will have a decrease in fever in approximately 1 week. The complaints of chest pain and sputum are expected to decrease in 4 weeks, and cough and shortness of breath will be greatly reduced in 6 weeks. Usually by 3 months, most of the symptoms will show improvement, but fatigue can last up to 6 months and most people return to their normal condition within 6 months [7].

## 4. Pathogenesis from post-acute COVID to organ dysfunction

The respiratory system is the most severely affected system during COVID-19 [16]. It is known that the disease, which has a largely asymptomatic or minimally symptomatic course, can be complicated by ARDS in the acute phase in some cases and may require intensive care. Immediately after the infection of the alveolar epithelium, thrombosis characterized by alveolar wall damage, edema, hyaline membranes, leukocyte infiltration and microangiopathic changes predominates, while proinflammatory cytokine release triggered by pattern recognition receptors and cell damage, and an uncontrolled inflammatory process may develop afterwards [17]. In addition to immune responses, especially kinin-kallikrein system, renin-angiotensin and coagulation system play an important role in the worsening of the clinical outcome [18]. In patients with ARDS, long periods of follow-up in the intensive care unit and mechanical ventilation might be required.

After the first four-week period, recovery can be achieved in most of the cases with severe disease, but unfortunately, organ damage can become permanent with prolonged symptoms, various pathologies and chronic processes. These post-acute complications include secondary infections, pulmonary function test disorders, pulmonary thromboembolism, pulmonary hypertension, and lung fibrosis [16].

Post-COVID secondary infections can change the course of recovery [19]. It is suggested that a dysregulation in immunological mechanisms develops and an immunosuppressive state occurs during the control of this response before it progresses to organ damage especially following the hyperinflammatory acute period that develops in COVID-19, which resembles the state of immunosuppression that develops after sepsis [8]. However, although these secondary infections play a role in complicating the clinical outcome, it is suggested that they are not associated with permanent organ dysfunction [20].

It has been reported that a decline in the measurements obtained in pulmonary function tests develop after COVID-19, which may continue for up to 12 months, and may even become permanent especially in cases with fibrosis or in relation to angiopathic changes [21–22]. It is stated that among the test variables, especially the decrease in carbon monoxide diffusion capacity is more prominent, which can be seen even in cases with normal lung volumes and can be a result of vascular pathologies. Respiratory muscle weakness, developing fibrosis, thrombosis and angiopathies, especially the ones associated with underlying diseases and intensive care follow-up processes, are some risk factors for the decline of pulmonary function [22–23].

It is known that vascular microangiopathic processes, micro and macro thrombosis are common from the early stages of COVID-19, and this condition is more common than in the other ARDS and viral infections [20]. It is known that the damage, inflammation and hypoxia that occurs during viral infection activates the coagulation cascade, and this process results in platelet activation with an increase in the level of mediators such as von Willebrand factor and P-selectin, as well as direct endothelial damage [24–26]. Increasing the complement system activation with the addition of factors such as antibody development may also contribute to the process [27]. It is seen that this hypercoagulability may continue in the post-acute period, and D-dimer levels, as an indicator of ongoing fibrin formation and degradation, can give an idea regarding this process. There are publications on cases diagnosed with pulmonary embolism in the post-acute and chronic period of infection [28–31]. Stasis, endothelial damage and hypercoagulability, the classical Virchow triad, might help understand the mechanism [32]. It can be estimated that the cases whose mobilization is impaired due to the ongoing disease state and symptoms in the post-acute period are especially at high risk and there is not enough information about when endothelial damage heals. There are comments that thrombomodulin levels can provide an information about the continuation of the endothelial damage [24]. It has been reported that inflammation continues in a prolonged period in some of the cases, and a prolonged inflammation state associated with IL-6 and Lipocalin-2 is observed [33–34]. It is suggested that hypercoagulability may be triggered continuously, especially by ongoing inflammation, and the antiphospholipid antibodies may also contribute to this situation [35]. However, since it is already known that transient antiphospholipid antibody positivity can be observed after infections, information about the prolonged period is far from certain, and further studies are needed [36].

Unfortunately, one of the most serious complications of the post-acute period is the development of fibrosis. It has been reported that fibrosis can be detected from the early period regardless of the pre-disease condition of the lung and the severity of the disease [17,37]. TGF-b, which is the most important factor in the healing process of the developing parenchymal damage and during the regression of inflammation, is thought to be the cytokine that plays the most important role in the progression to fibrosis in the post-COVID period [38–39]. The increase in TGF-b level leads to a decrease in ACE-2 expression and increase in Angiotensin-II levels that promotes proliferation of fibroblasts, migration to tissue, transformation into myofibroblasts, activation of myofibroblasts and ultimately extracellular matrix accumulation [38]. Since it is known that SARS-CoV-2, like other coronaviruses, increases TGF-b levels with a direct viral effect [40], it is understood that the development of fibrosis begins in the early stages, becomes aggravated by changes in angiotensin levels, and becomes evident during the resolution of inflammation. It has been suggested that fibrotic processes are more common especially in cases requiring mechanical ventilation in the early period, and this may be related to oxidative stress caused by high oxygen fractions and trauma caused by positive airway pressure, and these factors further facilitate the progression to fibrosis [38].

**Figure 1:**
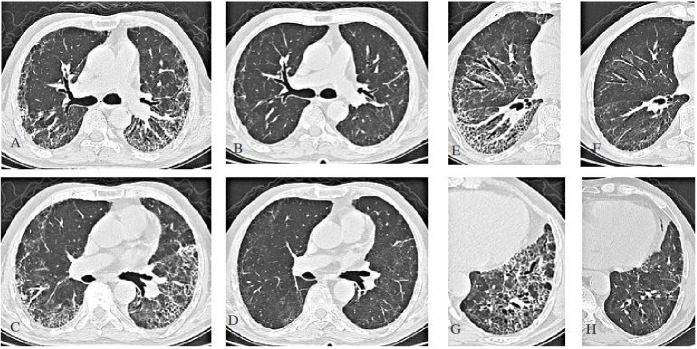
a-c-e-g: Chest CT of the patient who presented with dyspnea at 13 weeks post-COVID-19. b-d-f-h: Significant regression in CT findings of the patient are observed at the 25th week after steroid treatment. (From archives of Aydın Yılmaz)

The radiological findings obtained in this study and indicated in Figure 1 are as follows: Figures 1a, 1c, 1e, and 1g show the radiological findings of a patient with dyspnea at admission, whereas Figures 1b, 1d, 1f, and 1h show the radiological improvement of the same lung slices at 13 weeks post-COVID-19 after the patients received the corticosteroid treatment. (From archives of Ayd*ın Yılmaz*)

## 5. PCS clinical features

Post-COVID-19 syndrome is defined as the duration of symptoms and signs developing during or after COVID-19 infection for more than 12 weeks and exclusion of other reasons to explain this clinical condition. Symptoms and signs are manifested by frequently overlapping clusters that can fluctuate over time, change, and affect any system in the body [7]. Although the main route of entry of SARS-CoV-2 to the body is the upper and lower respiratory tract, it can cause diseases such as pneumonia, acute lung damage and endotheliitis, pulmonary fibrosis and thromboembolism in the long term, resulting in a decrease in quality of life. Advanced age, the presence of accompanying chronic diseases, and the severe course of acute COVID-19 disease increase the risk of developing post- COVID-19 syndrome Centers for Disease Control and Prevention. Post-COVID Conditions. Available from: https://www.cdc.gov/ coronavirus/2019-ncov/hcp/clinical-care [Accessed 06/2021]. It is estimated that 10%–35% of COVID-19 patients who do not require hospitalization develop post-covid symptoms regardless comorbidities, whereas in hospitalized patients and patients with serious illnesses this rate can be up to 80% [33]. Post-COVID syndrome classification was made by different centers according to initial symptoms, onset time and duration of symptoms together with organ dysfunctions [41–43]; however, standardization of the classification is needed to provide a common pathway for diagnostic and therapeutic, as well as research purposes.

The most common pulmonary symptoms in post-COVID syndrome are shortness of breath and cough [11, 44]. It has been reported that dyspnea and reduced exercise tolerance develop in 10%–40% of the hospitalized COVID-19 patients 2–4 months after discharge, and shortness of breath develops in 65% of patients hospitalized and discharged from the intensive care unit [45–46]. Commonly encountered clinical conditions are post-COVID interstitial lung disease (organized pneumonia, pulmonary fibrosis), pulmonary embolism and chronic cough, whereas cavitary lesions, small airway disease and development of pulmonary hypertension are mentioned as rare conditions. 

### 5.1. Chronic cough and its management

Cough, which is among the initial symptoms of acute COVID-19 disease, is a condition that is also frequently encountered in post-COVID syndrome, along with symptoms such as chronic fatigue, headache, and widespread pain. The prevalence of cough was reported as 7%–10% in two studies [47–48]. The reason of cough in some patients is not clear. However, unlike cough that persists after a cold or flu, chronic cough in post-COVID syndrome is often accompanied by other multisystemic manifestations that may indicate multifactorial pathogenesis or common mechanisms underlying these symptoms. Cough may result from invasion of vagal sensory neurons by SARS-CoV-2, or from a neuroinflammatory response, or both, and may lead to peripheral and central hypersensitivity in the cough pathways [49]. After the differential diagnosis of chronic cough is made (according to the guidelines), treatment can be started. However, although guidelines serve as a reference for current approaches to acute and chronic cough, the optimal management of cough associated with COVID-19 remains unclear.

In the NICE guideline, opioid-derived antitussives are recommended in acute COVID, but attention should be paid to their addictive potential and central nervous system side effects [7]. Oral or inhaled steroids can be given to asthmatic patients. Chronic refractory or unexplained cough in post-COVID syndrome may result from neuroinflammation leading to larynx and tracheal hypersensitivity. Neuromodulator agents such as gabapentin and pregabalin can be recommended. Antimuscarinic drugs such as tiotropium can be used to control COVID-19 cough as they can reduce cough sensitivity in acute viral upper respiratory tract infection. Speech and language therapy offered as part of a multimodal therapy in synergy with other aspects of pulmonary rehabilitation can help patients recover from post-COVID syndrome [49].

Studies are needed to provide insights into the epidemiology of post-COVID chronic cough and its impact on quality of life as well as cough hypersensitivity.

### 5.2. Cavitary lesions and management

The radiological specific findings of COVID-19 disease are unilateral/bilateral ground-glass opacities with peripheral distribution, fine reticular opacities, and vascular thickening [50]. The cavity is an air-filled space formed within the area of ​​pulmonary consolidation, mass, or nodule as a result of liquefaction of the necrotic portion of the lesion and evacuation of this necrotic material through the bronchial tree. It is rare for viral pneumonias (including SARS-CoV and MERS-CoV) to cause pulmonary cavitation even in severe and advanced viral infection [51]. However, cavitary lesions have been published as case series in the prolonged symptomatic period (4–12 weeks) in COVID-19 disease. In a series of 12 cases (1.7%) within the 689 COVID-19 pneumonia patient group reported by Zoumot Z et al., the mean time from the first symptom onset to the detection of cavitation was 36 (21–54) days, and all patients had bilateral peripheral ground glass opacities on their first chest tomography. Consolidation and air bronchograms were detected in most of the patients and cobblestone appearance with interlobular septal thickenings in half of them [52]. Tocilizumab and steroid therapy, which cause immunosuppression, were administered to patients followed up in the intensive care unit with severe COVID-19 pneumonia, but bacterial agents that could cause cavitary lesions were detected in only 4 patients. While simultaneous hemoptysis and pneumothorax developed in 2 patients, a total of 4 patients had in addition radiological findings compatible with invasive aspergillosis. The authors describe the development of cavitation in these patients as a result of multifactorial reasons such as: bacterial and fungal coinfection, immunosuppressive effects of glucocorticosteroids and tocilizumab, inflammatory pathways specific to SARS-CoV-2, susceptibility to COVID-19 related venous thromboembolism and micro-infarctions or infarctions causing cavitary lesions. They also emphasized that the natural course of these cavities in survivors is uncertain [52].

Tuberculosis disease, which is endemic in our country and usually presents with a cavity, has also been reported in several publications as a co-infection with COVID-19. Both diseases have common risk factors such as underlying comorbidities (diabetes, immunosuppression) and low socio-economic status, air pollution, living in crowded environments, and both of them are important infectious pathogens that continue to threaten the world population. Presence of active or previous history of tuberculosis has been found to be associated with the development of COVID-19 and might worsen the prognosis of acute infection [53].

It has been found that proinflammatory cytokines such as TNF-α, IL-6, IL-10, IL-1β and chemokines increase in COVID-19 patients (18). Increasing IL-10, Th1 suppression, and suppression of IL-6 level by tocilizumab used during the cytokine storm period increase the risk of invasive pulmonary aspergillosis, which progresses with a cavity and has a very poor prognosis in the follow-up period in COVID-19 patients [54–55].

Although there is no standard diagnosis or treatment algorithm in the management of cavitary lesions, which we encounter mostly in the prolonged symptomatic period in post-COVID syndrome, attention should be paid to fungal infections especially aspergillosis, tuberculosis, differential laboratory, and microbiological examinations should be performed and the appropriate treatment should be started as soon as possible.

**Figure 2:**
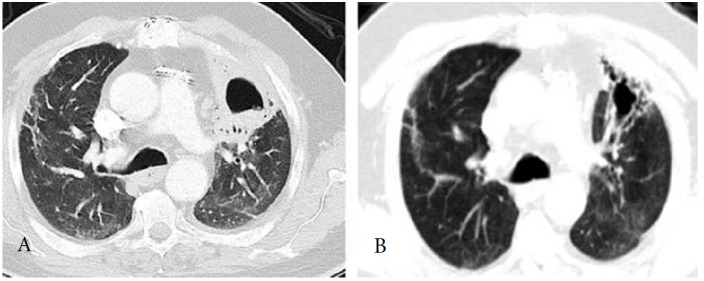
a: Chest CT of the patient who presented with a cavity in the left upper lobe at 12 weeks post-COVID. b: CT showed a regression of the cavity after 6 weeks of antibiotic treatment. (From archives of Aydın Yılmaz)

Below, in Figure 2a the CT findings of a patient presenting with a cavitary lesion are shown, and Figure 2b shows the remaining changes in lungs after antibiotic treatment. (From archives of Ayd*ın Yılmaz*)

### 5.3. Small airway disease and its management

The development of small airway disease (SAD) in post-COVID syndrome is a very new clinical condition. A study, which is still in the publication stage and included 100 patients, tried to show if there is a correlation between air trapping measured with quantitative thorax computed tomography (CT) and pulmonary function test [56]. Although it is difficult to define small airways on CT, studies have found that air trapping detected on CT can be a biomarker of functional SAD [57]. In this study, it was stated that SARS-CoV-2 infection itself causes functional SAD and air trapping, whereas restrictive lung disease and deterioration in gas exchange are caused by lung damage and ARDS, regardless of the underlying cause. 

The underlying mechanism is explained as follows:

The angiotensin-converting enzyme 2 (ACE2) receptor, which facilitates SARS-CoV-2 entry in cells, is expressed throughout the airway, including the small airways. Therefore, the functional SAD observed in patients in the post-COVID syndrome may result from direct infection of the small airways by SARS-CoV-2, even in patients with mild acute infection. In this case, post-COVID SAD may result from an ongoing damage-repair process, cellular debris, and/or abnormal mucus production. Conversely, the immune response induced by SARS-CoV-2 can directly induce post-COVID SAD even in the absence of infection. Regardless of the underlying mechanism, the development of functional SAD months after acute infection is of concern for airway remodeling and fibrosis.

In one study, no correlation was observed between pulmonary function tests, quantitative CT measurements of ground glass opacities, air trapping and the period time from diagnosis. The median time from diagnosis to pulmonary function measurements and chest CT imaging was approximately 75 days. Multicenter, prospective studies are needed for the frequency of SAD and treatment recommendations in post-COVID syndrome.

**Figure 3:**
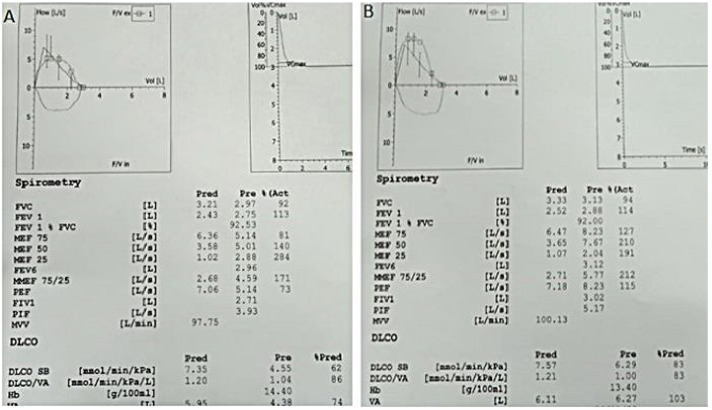
Diffusion and pulmonary function test findings of the patient above at first admission (a) and after steroid treatment (b). (From archives of Aydın Yılmaz)

Below, in Figure 3a, diffusion capacity and pulmonary function tests of a patient at admission, and, in Figure 3b, the improvement of these tests values after receiving corticosteroid treatment are shown. (From archives of Ayd*ın Yılmaz*)

### 5.4. Pulmonary hypertension and its management

Although the exact mechanism is not known in some viral infections such as HIV, the frequency of pulmonary arterial hypertension (PAH) has increased [58]. In the COVID-19 disease, the pulmonary artery wall thickness increased twice as compared to the H1N1-infected individuals as showed in autopsies. Pulmonary vascular thickening in COVID-19 is thought to have a vital role in the development of acute respiratory failure, and based on this result, we can think that patients recovering from COVID-19 may be prone to develop PAH and right heart failure [59–60].

## 6. COVID-19 and multisystem inflammatory syndrome in adults (MIS-A)

Multisystem inflammatory syndrome (MIS) is a finding that is observed in the prolonged course of COVID-19 but is not very common according to the available data. This syndrome, which was stated to affect children for the first time in articles published in March 2020 in England and the USA, was named as MIS-C (child) [61–63]. Shortly after MIS-C was defined (June 2020), a clinical picture compatible with MIS was reported also in adults and was defined as MIS-A(adult) [64–66]. Among the inflammatory syndromes secondary to COVID-19, Kawasaki-like syndrome has also been reported especially in children. Case reports that describe Kawasaki-like syndrome in adults are also available in the literature [67]. Most et al. also mention about the myocarditis-like syndrome in adults as another post-COVID inflammatory syndrome in their review [68]. A detailed Venn diagram of the overlap of symptoms and signs of Kawasaki disease, MIS, and acute cardiovascular syndrome is included in another part of this review. Here, only MIS-A will be discussed in details.

Cardiovascular, gastrointestinal, dermatological and neurological symptoms without serious respiratory disorders are reported in the first published cases with the diagnosis of MIS-A. In the course of MIS-A, diagnosis is challenging due to the heterogeneity of symptoms and findings. SARS-CoV-2 PCR or antigen test positivity may not be detected in the diagnosis of MIS-A, and antibody test positivity may be the only evidence that the case has COVID-19 disease [69].

The pathogenesis of the relationship between acute COVID-19 disease and MIS-A is not clearly understood, thus making it difficult to prevent the unwanted complications such as MIS-A. According to the data obtained from published case reports, the definition of MIS-A is made by the following five criteria [69]:

1. Presence of severe disease requiring hospitalization in an individual >21 years of age, 2. positive test result of SARS-CoV-2 infection (nucleic acid, antigen or antibody); 3. presence of severe dysfunction in one or more extrapulmonary organs/systems (hypotension or shock, cardiac dysfunction, arterial or venous thrombosis, acute liver injury), 4. elevated inflammatory parameters (CRP, ferritin, D-dimer or IL-6), 5. no serious respiratory problems (organ dysfunctions not associated with tissue hypoxia). MIS-A cannot be excluded in the presence of mild respiratory symptoms.

As of May 2021, as of the time of writing this article, the number of full-text articles accessed with the keyword “MIS-A and COVID-19” in the PubMed index was determined as 186. All of these articles were case series. These numbers indicate that MIS-A has been seen or reported relatively less, when compared to the numerous articles on COVID-19 published since the beginning of the pandemic.

Although hyperinflammation and extrapulmonary organ dysfunction are common in hospitalized adults with severe COVID-19, respiratory failure is often present in these severe cases. In MIS-A, there may be only mild respiratory symptoms, mild hypoxemia or rare radiological abnormalities. Although the pathophysiology of MIS is not fully known, it suggests that multi-organ/system involvement such as heart, brain, liver, kidney and gastrointestinal involvement is associated with endothelial damage, thromboinflammation, dysregulated immune responses, and renin-angiotensin-aldosterone system dysregulation [69].

The interval between COVID-19 infection and the development of MIS-A is not known. It may be a prolonged clinical manifestation of an acute infection or a postacute phenomenon. Dyspnea may become prominent on days 5–8 of the Covid-19 infection, whereas the critical illness occurs usually 10–12 days after symptom onset. On the other hand, MIS-A is reported usually within 2–5 weeks after infection.

In their review, Vogel et al. defined cases according to the levels of diagnostic certainty within four different groups as definitive, probable, possible case and case with insufficient evidence for MIS-C/A. This classification is a useful definition as it includes demographics, symptoms, signs and laboratory results [70].

Level 1: Definitive case

Age < 21 years (MIS-C) and ≥21 years (MIS-A); fever for ≥3 consecutive days; the presence of two or more of the following clinical features: mucocutaneous findings, gastrointestinal symptoms, shock/hypotension, neurological findings; elevation in any of inflammatory markers (CRP, ESR, ferritin or procalcitonin); the presence of two or more of disease activity markers: increased BNP or NT-pro BNP or troponin, neutrophilia, lymphopenia or thrombocytopenia, detection of cardiac involvement by echocardiography, EKG changes consistent with myocarditis or myo-pericarditis and the confirmation of active SARS-CoV-2 infection by laboratory or having a history of confirmed COVID-19 in the past 12 weeks or a history of close contact with a known case of COVID-19 in the last 12 weeks or a recent SARS-CoV-2 vaccination history.

Level 2: Probable case

Level 2a*: *Same criteria as Level 1, except for disease activity measurements; a history of known or strongly suspected COVID-19 in the last 12 weeks or a close contact with a known or strongly suspected COVID-19 case in the last 12 weeks or a recent SARS-CoV-2 vaccination.

Level 2b: Presence of the same criteria as Level 1 except fever that has lasted for 1–2 days or that can be subjective.

Level 3: Possible case

Level 3a: Age < 21 years (MIS-C) and ≥21 years (MIS-A); fever for >3 consecutive days; two or more of the following clinical features: mucocutaneous, gastrointestinal, shock/hypotension, neurological, physical examination findings of heart failure: gallop rhythm or rales, lower extremity edema, jugular venous engorgement, hepatosplenomegaly; absence of elevated inflammatory markers and a history of known or strongly suspected COVID-19 in the last 12 weeks or close contact with a known or strongly suspected COVID-19 case in the last 12 weeks or a recent SARS-CoV-2 vaccination.

Level 3b: Presence of the same criteria as Level 2a except fever that lasted for 1–2 days and that can be subjective.

Level 4: Insufficient evidence

A case that does not fully meet the case definitions in levels 1–3. 

The treatment for MIS-A is supportive, but there may also be a need for intensive care. The current protocol includes biological agents such as intravenous immunoglobulin (IVIG), corticosteroids, tocilizumab, anakinra or infliximab. It is reported that the treatment response is rapid, and the patient can improve dramatically within days. However, it may also result in unresponsiveness to treatment and death [71]. It is important for the clinician to notice the early findings and approach them in a proper way. Because it is a clinical condition that can be overlooked and antibody tests are not widely used yet, a careful and systematic approach is required for the diagnosis of MIS-A, with a good collaboration of multidisciplinary specialists.

## 7. PCS lung complications management

As more than one year has passed since the pandemics of SARS-CoV-2 started, we gained knowledge about the long-term pulmonary complications of COVID-19 disease. Experience with longer follow-ups will further shape the approach to PCS patients. Viral pneumonia is the most common and the most severe problem in the course of the disease. It is not yet clear whether disseminated COVID-19 pneumonia can have long-term effects resulting in persistent fibrosis in the lungs. The number of studies and reviews written on post-COVID pulmonary fibrosis is increasing day by day. Post-COVID pulmonary fibrosis is referred to as Post-COVID interstitial lung disease (PC-ILD) in most articles. In this article, the abbreviation PC-ILD will be used. In an article published from India in March 2021, PC-ILD was defined as ‘the tsunami that will follow the earthquake’ [38,72–73]. Although it is reported that there are three possible different clinical courses, the frequency of each is not known. According to this review, most lung involvements heal without sequelae, some heal with minimal fibrosis without worsening, and a few progress to advanced fibrosis that requires antifibrotic treatment. Advanced age, severe pneumonia, prolonged ICU stay and prolonged MV duration, smoking and chronic alcoholism history are some of the predictors for PC-ILD development. High CRP, IL-6 and LDH levels in the acute phase may lead to activation of fibroblast proliferation in the repair process of lung injury [72,73].

### 7.1. PC-ILD prevention and treatment

In order to minimize the risk factors for PC-ILD development shortening the length of stay in the intensive care unit, avoiding invasive mechanical ventilation support if possible, managing the case with low airway pressure in case of forced MV, proper management of concomitant diseases and prevention of bacterial superinfections are some of the measures that can be taken.

Treatment options can be listed as follows based on the available evidence [74]:

Antivirals: The close relationship between the development of lung fibrosis and high viral load is known, and the effectiveness of early antiviral treatments (remdesivir, favipiravir) to reduce viral load has been shown in some studies [74]. The necessity of effective antivirals in pandemic control is indisputable. However, there is no specific antiviral agent developed for the SARS-CoV-2 agent yet and many studies are ongoing in order to develop effective drugs.

Anti-inflammatory drugs: The efficacy of systemic steroids has been proven in the treatment of severe disease in the acute period. World Health Organization (WHO). Corticosteroids for COVID-19. Available from:https://www.who.int/publications/item/WHO-2019-nCoV-Corticosteroids-2020 [Accessed 06/2021]. However, there is insufficient evidence for long-term use in the prevention of PC-ILD. There are studies suggesting that 20–30 mg/day prednisolone should be started in the presence of diffuse ground glass opacities in the prevention of lung fibrosis and continued until radiological improvement is achieved [74].

Antifibrotics*:* As mentioned above, lung involvement should be monitored for up to 3 months in the post-COVID period, and it should be kept in mind that there may be a possibility for regression. In other words, it is not necessary to rush for the antifibrotic treatment. If fibrosis still persists at the end of the 12th week, there is a rationale for the use of these agents in the treatment of PC-ILD, although there is not enough data yet. Pirfenidone or nintedanib should be used for at least 1–3 months for a clear measurement of the antifibrotic response. In addition to clinical findings, response to antifibrotics should be evaluated objectively with HRCT, DLCO and 6MWT [75]. Concomitant use of steroids and antifibrotics is also being tried, and the long-term results are not yet known.

Pulmonary rehabilitation (PR): PR is a vital treatment approach that can also be applied at home. Since PC-ILD may also cause hypoxemia, long-term use of oxygen and PR at home can help in recovery [7].

Other supportive treatments: Anticoagulation, psychiatric support, family support, pneumococcal and influenza vaccinations are required until the patient is fully mobile, and evaluation of progressive and persistent PC-ILD for lung transplantation in selected cases is recommended [7].

### 7.2. Pulmonary embolism prevention and treatment

After the acute phase of the disease, the state of hypercoagulation may last for a long time, so long-term prophylaxis may be recommended in cases with severe disease. However, this issue is not very clear yet. In cases with severe disease and accompanying pulmonary embolism, anticoagulation administration is required according to the recommendations by the specific guidelines. It is recommended to evaluate the patient for the development of pulmonary hypertension (PHT) at the end of 12 weeks, and to discontinue the treatment at the end of the 3rd month if PHT has not developed. Lastly, the measurement and follow-up of D-Dimer level, which is also an acute phase reactant and is not specific for COVID-19, for chronic embolism is controversial [74]. T.C Sağlık Bakanlığı, COVID-19 enfeksiyonu antisitokin-antiinflamatuar tedaviler, koagülopati yönetimi. Available from: https://covid19.saglik.gov.tr [Accessed 06/2021]

**Figure 4:**
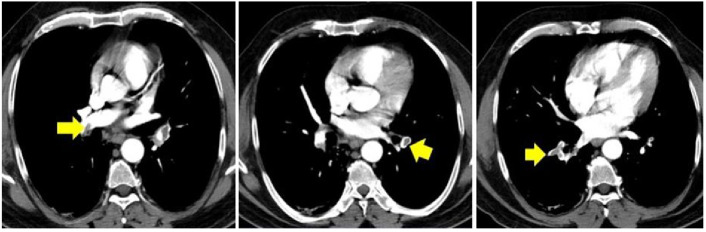
a-b-c: Chest CT of the patient who applied with diffuse chest pain and low oxygen saturation at 8weeks post-COVID: bilateral pulmonary embolism. (From archives of Aydın Yılmaz.

CT findings of a patient presenting with bilateral pulmonary embolism are shown in Figure 4. Figure 4a shows a centrally located thrombus on the right pulmonary artery, whereas Figure 4b and 4c show the presence of thrombus in segmental branches of the left and right pulmonary arteries, respectively. (From archives of Ayd*ın Yılmaz*)

## 8. Conclusion

COVID-19 disease is not just an acute infection, but is a complex entity with post-infection complications and long effects especially involving the pulmonary system, which emphasizes the fact that the treatment of this disease continues even after the patients have been discharged. Further detailed studies are needed in order to identify biomarkers and risk factors for the individuals that can progress, thus enabling a prompt intervention and treatment to minimize the long term effects. There are still many unknowns regarding the pathophysiology of this new disease, which makes the approach to these patients difficult for the moment and COVID-19 specialized hospitals or centers might help for an optimized treatment and follow-up.
